# Retraction Note: Periostin secreted by cancer-associated fibroblasts promotes cancer stemness in head and neck cancer by activating protein tyrosine kinase 7

**DOI:** 10.1038/s41419-024-06445-8

**Published:** 2024-01-15

**Authors:** Binbin Yu, Kailiu Wu, Xu Wang, Jianjun Zhang, Lizhen Wang, Yingying Jiang, Xueqin Zhu, Wantao Chen, Ming Yan

**Affiliations:** 1 Department of Oral and Maxillofacial-Head & Neck Oncology, Shanghai Ninth People’s Hospital & College of Stomatology, Shanghai Jiao Tong University School of Medicine, Shanghai, 200011 China; 2 National Clinical Research Center of Stomatology, Shanghai Key Laboratory of Stomatology & Shanghai Research Institute of Stomatology, Shanghai, 200011 China; 3 Department of Oral Pathology, Shanghai Ninth People’s Hospital, Shanghai Jiao Tong University School of Medicine, Shanghai, 200011 China

Retraction to: *Cell Death and Disease* 10.1038/s41419-018-1116-6, published online 22 October 2018

The Editors-in-Chief have retracted this article. Concerns were raised regarding figure 4H, specifically:

- The shNC Control panel appears to partially overlap with the shPTK7-1 rhPOSTN panel, but slightly resized.
- The SHPTK7-2 Control panel appears to partially overlap with the shPTK7-2 rhPOSTN panel, but slightly resized.

Author Ming Yan also stated that the authors found concerns with Figures 5D, 6A and 6D. Additionally, there appeared to be a number of discrepancies in the documentation regarding the ethics approval as provided by the authors. The Editors-in-Chief therefore no longer has confidence in the results and conclusions of this article.

Author Ming Yan has stated that the authors disagree with this retraction.

